# The causal effects of health conditions and risk factors on social and socioeconomic outcomes: Mendelian randomization in UK Biobank

**DOI:** 10.1093/ije/dyaa114

**Published:** 2020-08-18

**Authors:** Sean Harrison, Alisha R Davies, Matt Dickson, Jessica Tyrrell, Michael J Green, Srinivasa Vittal Katikireddi, Desmond Campbell, Marcus Munafò, Padraig Dixon, Hayley E Jones, Frances Rice, Neil M Davies, Laura D Howe

**Affiliations:** 1 MRC Integrative Epidemiology Unit (IEU), Bristol Medical School, University of Bristol, Bristol, UK; 2 Population Health Sciences, Bristol Medical School, University of Bristol, Bristol, UK; 3 Research and Evaluation Division, Public Health Wales NHS Trust, Cardiff, UK; 4 Institute for Policy Research, University of Bath, Bath, UK; 5 University of Exeter Medical School, RILD Building, RD&E Hospital Wonford, Exeter, UK; 6 MRC/CSO Social and Public Health Sciences Unit, University of Glasgow, Glasgow, UK; 7 UK Centre for Tobacco and Alcohol Studies, School of Experimental Psychology, University of Bristol, Bristol, UK; 8 Medical Research Council Centre for Neuropsychiatric Genetics and Genomics, Division of Psychological Medicine and Clinical Neurosciences, Cardiff University, Cardiff, UK; 9 K.G. Jebsen Center for Genetic Epidemiology, Department of Public Health and Nursing, Norwegian University of Science and Technology, Norway

**Keywords:** Health, socioeconomic, social, economic, health risk factors, health conditions, Mendelian randomization, UK Biobank

## Abstract

**Background:**

We aimed to estimate the causal effect of health conditions and risk factors on social and socioeconomic outcomes in UK Biobank. Evidence on socioeconomic impacts is important to understand because it can help governments, policy makers and decision makers allocate resources efficiently and effectively.

**Methods:**

We used Mendelian randomization to estimate the causal effects of eight health conditions (asthma, breast cancer, coronary heart disease, depression, eczema, migraine, osteoarthritis, type 2 diabetes) and five health risk factors [alcohol intake, body mass index (BMI), cholesterol, systolic blood pressure, smoking] on 19 social and socioeconomic outcomes in 336 997 men and women of White British ancestry in UK Biobank, aged between 39 and 72 years. Outcomes included annual household income, employment, deprivation [measured by the Townsend deprivation index (TDI)], degree-level education, happiness, loneliness and 13 other social and socioeconomic outcomes.

**Results:**

Results suggested that BMI, smoking and alcohol intake affect many socioeconomic outcomes. For example, smoking was estimated to reduce household income [mean difference = -£22 838, 95% confidence interval (CI): -£31 354 to -£14 321] and the chance of owning accommodation [absolute percentage change (APC) = -20.8%, 95% CI: -28.2% to -13.4%], of being satisfied with health (APC = -35.4%, 95% CI: -51.2% to -19.5%) and of obtaining a university degree (APC = -65.9%, 95% CI: -81.4% to -50.4%), while also increasing deprivation (mean difference in TDI = 1.73, 95% CI: 1.02 to 2.44, approximately 216% of a decile of TDI). There was evidence that asthma decreased household income, the chance of obtaining a university degree and the chance of cohabiting, and migraine reduced the chance of having a weekly leisure or social activity, especially in men. For other associations, estimates were null.

**Conclusions:**

Higher BMI, alcohol intake and smoking were all estimated to adversely affect multiple social and socioeconomic outcomes. Effects were not detected between health conditions and socioeconomic outcomes using Mendelian randomization, with the exceptions of depression, asthma and migraines. This may reflect true null associations, selection bias given the relative health and age of participants in UK Biobank, and/or lack of power to detect effects.

**Key Messages**

- Studies have shown associations between poor health and adverse social (e.g. well-being, social contact) and socioeconomic (e.g. educational attainment, income, employment) outcomes, but there is also strong evidence that social and socioeconomic factors influence health.
- These bidirectional relationships, as well as confounding, make it difficult to establish whether health conditions and health risk factors have causal effects on social and socioeconomic outcomes.
- Mendelian randomization is a technique that uses genetic variants robustly related to an exposure of interest (here, health conditions and risk factors for poor health) as a proxy for the exposure, and is typically less prone to both reverse causation and confounding, allowing us to estimate more causal effects of health conditions and risk factors on social and socioeconomic outcomes.
- This study suggests causal effects of higher body mass index, smoking and alcohol use on a range of social and socioeconomic outcomes, implying that population-level improvements in these risk factors may, in addition to the well-known health benefits, have social and socioeconomic benefits for individuals and society.
- There was evidence that: asthma increased deprivation and decreased household income and the chance of having a university degree; depression increased loneliness and decreased happiness; and migraine reduced the chance of having a weekly leisure or social activity, especially in men. There was little evidence for causal effects of cholesterol, systolic blood pressure or breast cancer on any social and socioeconomic outcome.

## Introduction

Poor health has the potential to affect an individual’s ability to engage with society.^1–4^ For example, illnesses or adverse health behaviours could influence the ability to attend and concentrate at school or at work and hence affect educational attainment, employment and income. Illness and health behaviours may also affect an individual’s ability to maintain well-being and an active social life. From an individual perspective, maintaining good health can therefore have considerable social and socioeconomic benefits.^5^ Similarly from a population perspective, improving population health could lead to a happier and more productive population.^6^

Understanding the causal impacts of health on social and socioeconomic outcomes can help demonstrate the potential broader benefits of investing in effective health policy, thereby strengthening the case for cross-governmental action to improve health and its wider determinants at the population level.^7^ Furthermore, patients require accurate information about how their lives might be affected by their health, for example on returning to work after cancer.^8^ However, studying the social and socioeconomic consequences of ill health (‘social drift’) is challenging because of social causation, i.e. the strong role of social and socioeconomic circumstances in disease causation. Social causation means that associations between health and social and socioeconomic outcomes are likely to be severely biased by confounding and reverse causality. Methodological approaches strengthening causal inference in this field are therefore essential.

Mendelian randomization is a technique that uses genetic variants robustly related to an exposure of interest (here, health conditions and risk factors for poor health) as proxies for the exposure (instrumental variables).^9^^,^^10^ Since genetic variants are randomly allocated at conception, conditional on parental genotypes, results from Mendelian randomization studies are much less likely to suffer from confounding and reverse causality than traditional observational studies.^11^ In this paper, we apply Mendelian randomization within a large study of UK individuals aged between 39 and 72 years, to estimate the causal effects of health conditions and risk factors with the greatest burden on UK adults on a range of social (e.g. social contact, well-being and cohabitation status) and socioeconomic (e.g. education, employment, income) outcomes.

## Methods

### Population

UK Biobank is a population-based health research resource consisting of approximately 500 000 people, who were recruited between the years 2006 and 2010 from 22 centres across the UK.^12^ Participants provided medical history and socioeconomic information via questionnaires, interviews and anthropometric measures at recruitment. Medical data from hospital episode statistics (HES) and the cancer registry have been linked to participants. The study design, participants and quality control methods have been described in detail previously.^13–15^ UK Biobank received ethics approval from the Research Ethics Committee (REC reference for UK Biobank is 11/NW/0382).

We restricted analyses to unrelated individuals of White British ancestry. Full details of inclusion criteria and genotyping are in Supplementary Information Section 1, available as Supplementary data at *IJE* online. After exclusions, 336 997 participants remained in the dataset.

### Measures of health conditions and risk factors (exposures) 

We used the Global Burden of Disease Study 2010^16^ to identify health conditions and risk factors that contributed 100 or more disability-adjusted life years lost per 100 000 adults in the UK. From this list, we restricted our analysis to health conditions and risk factors with known genetic determinants and a prevalence of ≥2% among UK Biobank participants. This resulted in the inclusion of eight health conditions: asthma, breast cancer, coronary heart disease, depression, eczema, migraine, osteoarthritis and type 2 diabetes; and five risk factors: alcohol consumption, body mass index (BMI), cholesterol, smoking and systolic blood pressure (Supplementary Figure 1, and Supplementary Table 1, available as Supplementary data at *IJE* online).

**Figure 1:**
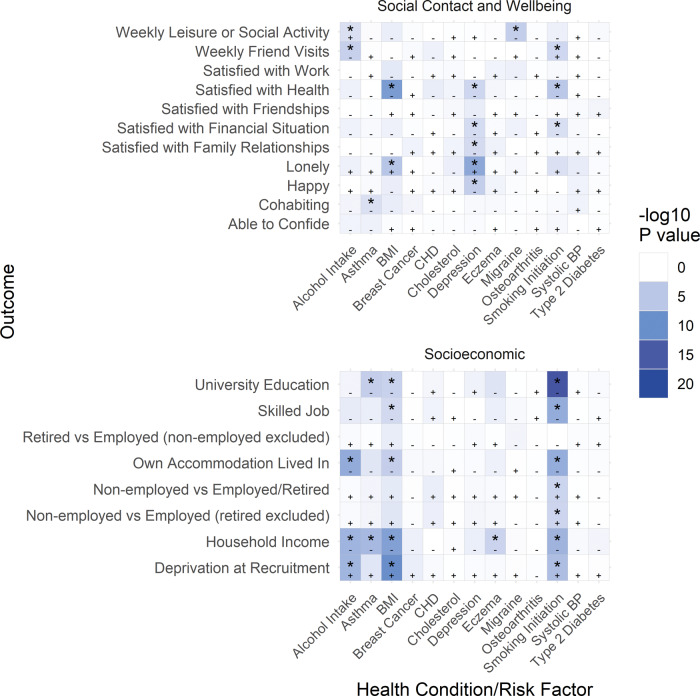
Heat map of results from the main analysis. Each cell shows the *P*-value of the main analysis result for the indicated exposure and outcome, with the colour of the cell increasing in intensity as the *P-*value of the analysis decreases. Starred results are below the Bonferroni-corrected *P*-value threshold (*P* <0.0026), negative effect directions are denoted with a minus symbol (-) and positive effect directions are denoted with a plus symbol (+).

**Table 1.** Summary demographics

Variable	All	*N*	Men	*N*	Women	*N*
*N*	336 997		155 714		181 283	
Age at recruitment, years [mean (SD)]	56.9 (8.00)	336 997	57.1 (8.09)	155 714	56.7 (7.91)	181 283
Health conditions						
Asthma [*N* (%)]	42 832 (12.71)	336 997	18 333 (11.77)	155 714	24 499 (13.51)	181 283
Breast cancer [*N* (%)]	7625 (2.26)	336 997	74 (0.05)	155 714	7551 (4.17)	181 283
Coronary heart disease [*N* (%)]	16 055 (4.76)	336 997	11 351 (7.29)	155 714	4704 (2.59)	181 283
Depression^a^ [*N* (%)]	19 088 (20.28)	94 131	6841 (15.14)	45 184	12 247 (25.02)	48 947
Eczema [*N* (%)]	8685 (2.58)	336 997	3961 (2.54)	155 714	4724 (2.61)	181 283
Migraine [*N* (%)]	10 603 (3.15)	336 997	2359 (1.51)	155 714	8244 (4.55)	181 283
Osteoarthritis [*N* (%)]	36 683 (10.89)	336 997	14 404 (9.25)	155 714	22 279 (12.29)	181 283
Type 2 diabetes^b^ [*N* (%)]	15 140 (4.51)	335 454	9349 (6.04)	154 820	5791 (3.21)	180 634
Risk factors						
Alcohol intake per week, units of alcohol [mean (SD)]	18.8 (16.51)	252 578	23.7 (18.76)	126 820	13.8 (12.00)	125 758
Body mass index, kg/m^2^ [mean (SD)]	27.4 (4.75)	335 916	27.8 (4.22)	155 193	27.0 (5.13)	180 723
Cholesterol, mmol/l [mean (SD)]	5.7 (1.14)	321 282	5.5 (1.13)	148 546	5.9 (1.13)	172 736
Ever smoked [*N* (%)]	98 996 (29.48)	335 829	51 812 (33.39)	155 154	47 184 (26.12)	180 675
Lifetime tobacco smoking [mean (SD)]	0.3 (0.68)	335 829	0.4 (0.72)	155 154	0.3 (0.63)	180 675
Systolic blood pressure, mmHg [mean (SD)]	140.2 (19.66)	336 684	143.2 (18.52)	155 633	137.6 (20.24)	181 051
Outcomes						
Socioeconomic						
Average total household income before tax [mean (SD)]	£44 409 (33 181)	290 457	£46 508 (34 101)	139 975	£42 458 (32 179)	150 482
<£18 000 [*N* (%)]	63 004 (21.69)	63 004	27 238 (19.46)	27 238	35 766 (23.77)	35 766
£18 000 to £30 999 [*N* (%)]	74 531 (25.66)	74 531	34 451 (24.61)	34 451	40 080 (26.63)	40 080
£31 000 to £51 999 [*N* (%)]	76 966 (26.50)	76 966	38 213 (27.30)	38 213	38 753 (25.75)	38 753
£52 000 to £100 000 [*N* (%)]	60 264 (20.75)	60 264	31 615 (22.59)	31 615	28 649 (19.04)	28 649
>£100 000 [*N* (%)]	15 692 (5.40)	15 692	8458 (6.04)	8458	7234 (4.81)	7234
Townsend Deprivation Index (TDI) at recruitment [mean (SD)]	−1.6 (2.93)	336 600	−1.5 (2.99)	155 531	−1.6 (2.88)	181 069
Non-employed [*N* (%)]	25 448 (7.61)	334 514	10 139 (6.56)	154 556	15 309 (8.51)	179 958
Non-employed (retired excluded) [*N* (%)]	25 448 (11.77)	216 241	10 139 (9.83)	103 134	15 309 (13.53)	113 107
Retired [*N* (%)]	118 273 (38.27)	309 066	51 422 (35.61)	144 417	66 851 (40.60)	164 649
Skilled job [*N* (%)]	181 138 (82.60)	219 290	88 921 (84.16)	105 656	92 217 (81.15)	113 634
Degree-level education [*N* (%)]	106 750 (38.57)	276 784	51 813 (40.48)	127 983	54 937 (36.92)	148 801
Own accommodation lived in [*N* (%)]	304 492 (91.47)	332 904	139 559 (90.79)	153 708	164 933 (92.04)	179 196
Social						
Able to confide (weekly or more frequently) [*N* (%)]	245 029 (74.84)	327 392	107 078 (70.99)	150 834	137 951 (78.13)	176 558
Frequency of friend/family visits (weekly or more frequently) [*N* (%)]	264 355 (78.90)	335 071	114 730 (74.17)	154 690	149 625 (82.95)	180 381
Cohabiting [*N* (%)]	249 951 (74.55)	335 271	120 960 (78.09)	154 895	128 991 (71.51)	180 376
Leisure/social activity [*N* (%)]	234 303 (69.70)	336 170	108 670 (69.96)	155 338	125 633 (69.47)	180 832
Lonely or isolated [*N* (%)]	58 573 (17.64)	332 073	22 158 (14.43)	153 549	36 415 (20.40)	178 524
Happy [*N* (%)]	106 155 (95.67)	110 958	49 098 (95.23)	51 556	57 057 (96.05)	59 402
Satisfied with family relationship [*N* (%)]	103 620 (93.93)	110 312	47 863 (93.59)	51 143	55 757 (94.23)	59 169
Satisfied with financial situation [*N* (%)]	96 704 (87.24)	110 843	44 488 (86.37)	51 507	52 216 (88.00)	59 336
Satisfied with friendships [*N* (%)]	106 855 (97.01)	110 144	49 012 (96.13)	50 985	57 843 (97.78)	59 159
Satisfied with health [*N* (%)]	96 555 (86.99)	110 997	44 828 (86.87)	51 602	51 727 (87.09)	59 395
Satisfied with work/job [*N* (%)]	68 536 (91.05)	75 277	31 898 (89.60)	35 602	36 638 (92.35)	39 675

SD, standard deviation.

a Depression was restricted to participants recruited to the 10 UK BIobank centres that asked questions related to depression, and who were not in the pilot sample.

b Participants with type 1 diabetes were excluded from all type 2 diabetes analyses.

Except for depression, we categorized a participant as having a health condition if they reported the condition at the baseline visit, or if they had the corresponding HES or cancer registry ICD-9 or ICD-10 code for the health condition before the baseline visit (ICD codes and specific questions used shown in Supplementary Table 1, available as Supplementary data at *IJE* online).

We coded depression as in Tyrrell *et al*. (2018),^17^ where participants were considered to have depression if they self-reported seeing a GP or psychiatrist for nerves, anxiety or depression and reported at least a 2-week duration of depression or unenthusiasm, or had the relevant ICD-9 or ICD-10 codes for depression. Participants were considered to not have depression if they did not report ever visiting a GP or psychiatrist for nerves, anxiety or depression, did not self-report having depression and did not have an ICD code for depression. Only 10 centres asked the questions related to depression, so only participants from these centres were considered in the depression analyses. The measurement of health risk factors is described in Box 1.

**Box 1 Measurement of health risk factors at baseline**

Alcohol intake

We estimated the average weekly intake of alcoholic units (10 ml of pure alcohol) for all participants based on the average reported intake of six different types of alcoholic beverage. The nominal number of units we assigned per drink for each type of alcoholic beverage is listed below:

- red wine: 125 ml (6/bottle), 14% = 1.75 units
- champagne/white wine: 125 ml (6/bottle), 14% = 1.75 units
- beer/cider: 1 pint, 3.5% = 2 units
- spirits: 25 ml (25 standard measures in a normal sized bottle), 40% = 1 unit
- fortified wine: 60 ml (12/bottle), 20% = 1.2 units
- other: unknown, example is an alcopop = 1 unit

We removed self-reported former drinkers, participants with a very high number of units per week (>200 units), and participants who did not report they were never drinkers but who answered none of the questions about weekly alcohol intake, leaving 252 585 participants (75%).

Body mass index

BMI was estimated as measured weight in kilograms divided by measured height in metres squared.

Cholesterol

Cholesterol was measured by UK Biobank at baseline (measured by CHO-POD analysis on a Beckman Coulter AU5800).

Smoking

We used two measures of self-reported smoking:

Lifetime smoking index: a composite (continuous) measure of relevant smoking variables with a simulated half-time constant representing the decreasing effect of smoking on health outcomes over time. This variable was created by Wootton *et al*. and used in a paper studying smoking and depression/schizophrenia.^18^

Smoking initiation: a binary measure indicating whether participants had ever versus never smoked, based on whether the lifetime smoking index value had a non-zero value.

Systolic blood pressure

Systolic blood pressure was measured using an automated device, and two measurements were taken a few moments apart. If the standard automated device could not be employed, two manual readings were taken instead.

### Polygenic risk scores (instrumental variables)

We searched previous genome-wide association studies (GWASs) for single nucleotide polymorphisms (SNPs) with strong evidence of associations for each health condition and risk factor, defined as having a *P*-value at genome-wide significance (P ≤ 5 × 10^–8^) (further details in Supplementary Information Section 2 and Supplementary Tables 2 and 3, available as Supplementary data at *IJE* online). The polygenic risk scores (PRSs) for each health condition and risk factor were then calculated as the sum of the effect alleles for all SNPs associated with the health condition or risk factor, with each SNP weighted by the regression coefficient from the GWAS from which the SNP was identified.

**Table 2.** Main MR, split-sample MR and multivariable-adjusted analysis results for all outcomes where the main or split-sample Mendelian randomization analysis had a *P*-value less than 0.0026

Health condition/risk factor	Outcome	*N*	Main MR analysis		Split-sample MR analysis		Multivariable adjusted analysis		*P* for endogeneity
			Beta (95% CI)	*P*	Beta (95% CI)	*P*	Beta (95% CI)	*P*	
Alcohol intake (5 units/week)	Cohabiting	251 512	−0.8% (-1.9% to 0.3%)	1.41E-01	−1.5% (-2.4% to -0.6%)	1.56E-03	−0.4% (-0.4% to -0.3%)	0.00E+00	4.30E-01
Alcohol intake (5 units/week)	Household income	219 924	£-2446 (£-3362 to £-1530)	1.65E-07	£-509 (£-1264 to £247)	1.87E-01	£442 (£400 to £484)	0.00E+00	1.54E-10
Alcohol intake (5 units/week)	Own accommodation lived in	249 886	−1.8% (-2.4% to -1.2%)	2.34E-08	−1.2% (-1.7% to -0.6%)	2.65E-05	−0.2% (-0.2% to -0.1%)	4.30E-25	2.24E-07
Alcohol intake (5 units/week)	TDI at recruitment	252 287	0.18 (0.11 to 0.25)	2.56E-07	0.14 (0.08 to 0.19)	3.66E-06	0.03 (0.03 to 0.04)	0.00E+00	1.78E-05
Alcohol intake (5 units/week)	Weekly friend visits	251 264	−2.1% (-3.2% to -1.1%)	7.91E-05	−1.1% (-2.0% to -0.2%)	1.24E-02	−0.0% (-0.1% to 0.0%)	8.79E-02	9.03E-05
Alcohol intake (5 units/week)	Weekly leisure or social activity	252 094	1.8% (0.7% to 3.0%)	2.10E-03	0.2% (-0.8% to 1.1%)	7.41E-01	0.8% (0.8% to 0.9%)	0.00E+00	9.61E-02
Asthma	Cohabiting	335 271	−11.0% (-17.9% to -4.0%)	2.08E-03	−3.0% (-7.7% to 1.6%)	1.96E-01	−1.6% (-2.0% to -1.1%)	1.76E-12	8.15E-03
Asthma	Household income	290 457	£-13 474 (£-18 749 to £-8199)	5.54E-07	£-6462 (£-9927 to £-2996)	2.58E-04	£-693 (£-1029 to £-357)	5.35E-05	1.57E-06
Asthma	University education	276 784	−17.1% (-25.4% to -8.7%)	5.83E-05	−9.3% (-14.7% to -3.9%)	7.37E-04	2.1% (1.6% to 2.7%)	1.20E-14	4.87E-06
Body mass index (5 kg/m^2^)	Household income	289 594	£-2777 (£-3692 to £-1863)	2.65E-09	£-4169 (£-4982 to £-3356)	9.15E-24	£-2332 (£-2451 to £-2212)	0.00E+00	3.36E-01
Body mass index (5 kg/m^2^)	Lonely	331 018	2.4% (1.4% to 3.5%)	7.06E-06	3.3% (2.3% to 4.2%)	1.23E-11	2.7% (2.6% to 2.9%)	0.00E+00	5.53E-01
Body mass index (5 kg/m^2^)	Non-employed vs employed (retired excluded)	215 625	1.4% (0.4% to 2.5%)	5.66E-03	2.3% (1.3% to 3.2%)	1.58E-06	1.9% (1.8% to 2.0%)	0.00E+00	3.75E-01
Body mass index (5 kg/m^2^)	Non-employed vs employed/retired	333 446	0.9% (0.2% to 1.6%)	1.34E-02	1.5% (0.8% to 2.1%)	8.27E-06	1.3% (1.2% to 1.4%)	0.00E+00	2.55E-01
Body mass index (5 kg/m^2^)	Own accommodation lived in	331 854	−1.6% (-2.4% to -0.8%)	5.75E-05	−3.1% (-3.8% to -2.4%)	2.03E-18	−2.6% (-2.7% to -2.5%)	0.00E+00	9.44E-03
Body mass index (5 kg/m^2^)	Satisfied with financial situation	110 373	−1.6% (-3.2% to 0.0%)	5.41E-02	−3.1% (-4.5% to -1.6%)	3.72E-05	−3.4% (-3.6% to -3.2%)	0.00E+00	2.63E-02
Body mass index (5 kg/m^2^)	Satisfied with health	110 513	−5.2% (-6.8% to -3.5%)	9.18E-10	−6.7% (-8.1% to -5.2%)	1.05E-18	−7.6% (-7.8% to -7.4%)	0.00E+00	3.53E-03
Body mass index (5 kg/m^2^)	Skilled job	218 811	−2.3% (-3.5% to -1.0%)	4.52E-04	−4.5% (-5.7% to -3.4%)	5.23E-15	−2.3% (-2.5% to -2.1%)	0.00E+00	9.41E-01
Body mass index (5 kg/m^2^)	TDI at recruitment	335 524	0.25 (0.17 to 0.33)	8.25E-11	0.35 (0.29 to 0.42)	7.44E-25	0.29 (0.28 to 0.30)	0.00E+00	2.94E-01
Body mass index (5 kg/m^2^)	University education	276 021	−2.9% (-4.4% to -1.5%)	9.41E-05	−7.2% (-8.6% to -5.9%)	5.03E-27	−5.4% (-5.5% to -5.2%)	0.00E+00	1.12E-03
Body mass index (5 kg/m^2^)	Weekly leisure or social activity	335 096	−1.4% (-2.7% to -0.1%)	2.89E-02	−3.0% (-4.2% to -1.9%)	1.87E-07	−2.6% (-2.8% to -2.5%)	0.00E+00	5.51E-02
Depression	Happy	69 646	−19.1% (-28.4% to -9.8%)	5.33E-05			−7.4% (-7.7% to -7.0%)	0.00E+00	9.81E-03
Depression	Lonely	92 977	58.7% (38.5% to 78.9%)	1.19E-08			18.4% (17.8% to 19.0%)	0.00E+00	1.93E-05
Depression	Satisfied with family relationships	69 271	−19.3% (-30.4% to -8.1%)	7.42E-04			−6.9% (-7.2% to -6.5%)	0.00E+00	2.56E-02
Depression	Satisfied with financial situation	69 554	−26.4% (-41.9% to -10.9%)	8.14E-04			−9.1% (-9.7% to -8.6%)	0.00E+00	2.45E-02
Depression	Satisfied with health	69 650	−29.1% (-44.6% to -13.6%)	2.28E-04			−11.1% (-11.6% to -10.5%)	0.00E+00	1.84E-02
Eczema	Household income	290 457	£-46 965 (£-71 028 to £-22 902)	1.31E-04	£-12 545 (£-30 268 to £5177)	1.65E-01	£158 (£-544 to £859)	6.60E-01	7.42E-05
Lifetime smoking (SD)	Cohabiting	334 122			−5.4% (-8.8% to -2.0%)	1.74E-03	−4.5% (-4.7% to -4.4%)	0.00E+00	
Lifetime smoking (SD)	Household income	289 728			£-7585 (£-10 155 to £-5014)	7.31E-09	£-4088 (£-4201 to £-3976)	0.00E+00	
Lifetime smoking (SD)	Non-employed vs employed (retired excluded)	215 665			5.9% (2.9% to 8.9%)	1.41E-04	4.1% (4.0% to 4.2%)	0.00E+00	
Lifetime smoking (SD)	Non-employed vs employed/retired	333 384			4.2% (2.1% to 6.2%)	9.42E-05	2.7% (2.6% to 2.8%)	0.00E+00	
Lifetime smoking (SD)	Own accommodation lived in	331 776			−8.6% (-10.7% to -6.4%)	8.83E-15	−5.1% (-5.2% to -5.0%)	0.00E+00	
Lifetime smoking (SD)	Satisfied with financial situation	110 501			−10.0% (-14.7% to -5.3%)	2.75E-05	−4.0% (-4.2% to -3.8%)	0.00E+00	
Lifetime smoking (SD)	Satisfied with health	110 649			−8.4% (-13.3% to -3.6%)	5.88E-04	−3.7% (-3.9% to -3.5%)	0.00E+00	
Lifetime smoking (SD)	Skilled job	218 695			−8.6% (-12.7% to -4.5%)	4.31E-05	−4.0% (-4.2% to -3.9%)	0.00E+00	
Lifetime smoking (SD)	TDI at recruitment	335 434			0.98 (0.76 to 1.19)	4.42E-19	0.53 (0.52 to 0.54)	0.00E+00	
Lifetime smoking (SD)	University education	276 177			−15.9% (-20.7% to -11.1%)	7.32E-11	−6.0% (-6.2% to -5.8%)	0.00E+00	
Migraine	Weekly leisure or social activity	336 170	−47.9% (-71.1% to -24.7%)	5.15E-05	−26.3% (-57.7% to 5.2%)	1.01E-01	−2.9% (-3.8% to -2.0%)	2.02E-10	1.12E-04
Smoking initiation	Household income	289 728	£-22 838 (£-31 354 to £-14 321)	1.47E-07	£-18 545 (£-25 572 to £-11 519)	2.30E-07	£-5974 (£-6220 to £-5728)	0.00E+00	6.24E-05
Smoking initiation	Non-employed vs employed (retired excluded)	215 665	19.0% (9.0% to 29.0%)	2.07E-04	10.0% (1.7% to 18.3%)	1.87E-02	5.3% (5.0% to 5.6%)	0.00E+00	6.62E-03
Smoking initiation	Non-employed vs employed/retired	333 384	13.3% (6.3% to 20.2%)	1.78E-04	6.4% (0.9% to 11.9%)	2.28E-02	3.6% (3.4% to 3.7%)	0.00E+00	5.35E-03
Smoking initiation	Own accommodation lived in	331 776	−20.8% (-28.2% to -13.4%)	3.90E-08	−16.3% (-22.1% to -10.5%)	3.13E-08	−6.7% (-6.9% to -6.5%)	0.00E+00	1.27E-04
Smoking initiation	Satisfied with financial situation	110 501	−23.6% (-38.5% to -8.7%)	1.86E-03	−22.7% (-36.0% to -9.4%)	8.22E-04	−5.7% (-6.2% to -5.3%)	0.00E+00	1.50E-02
Smoking initiation	Satisfied with health	110 649	−35.4% (-51.2% to -19.5%)	1.21E-05	−22.4% (-36.0% to -8.9%)	1.20E-03	−5.6% (-6.0% to -5.2%)	0.00E+00	7.13E-05
Smoking initiation	Skilled job	218 695	−37.0% (-50.0% to -23.9%)	2.96E-08	−14.1% (-24.3% to -3.9%)	6.61E-03	−5.5% (-5.9% to -5.2%)	0.00E+00	5.00E-07
Smoking initiation	TDI at recruitment	335 434	1.73 (1.02 to 2.44)	1.69E-06	2.26 (1.68 to 2.84)	2.41E-14	0.79 (0.77 to 0.81)	0.00E+00	8.58E-03
Smoking initiation	University education	276 177	−65.9% (-81.4% to -50.4%)	9.01E-17	−35.7% (-47.1% to -24.2%)	9.56E-10	−9.7% (-10.1% to -9.3%)	0.00E+00	1.19E-15
Smoking initiation	Weekly friend visits	333 955	19.8% (9.2% to 30.5%)	2.70E-04	−2.8% (-11.2% to 5.6%)	5.13E-01	−0.4% (-0.7% to -0.1%)	4.44E-03	1.32E-04

MR, Mendelian randomization; SD, standard deviation; vs, versus.

### Covariates

Age, sex and UK Biobank recruitment centre were reported at the baseline assessment, and genetic principal components (used to control for population stratification^19^) were derived by UK Biobank.

### Social and socioeconomic measures (outcomes)

We selected social and socioeconomic outcomes measured at the UK Biobank baseline assessment centre. Where possible, we dichotomized outcomes to simplify interpretability and comparability across outcomes. Box 2 contains a list of all outcomes; Supplementary Information Section 3, and Supplementary Table 4, available as Supplementary data at *IJE* online, give further information on how each outcome was measured.

We considered breast cancer, coronary heart disease, osteoarthritis, cholesterol or systolic blood pressure unlikely to have plausible causal effects on the chance of obtaining a university degree, given that these health conditions usually occur later in life; the Mendelian randomization effect estimates for these associations were thus used as negative controls (i.e. where no effect should be expected).^20^^,^^21^

**Box 2 List of all social and socioeconomic measures (outcomes)**

*Socioeconomic outcomes*
 

- Average household income before tax, with each category assigned the mid-point of the range (and open-ended categories a nominal value) to allow for continuous analysis:*
  - <£18 000 = £15 000
  - £18 000 to £30 999 = £24 500
  - £31 000 to £51 999 = £41 500
  - £52 000 to £100 000 = £76 000
  - >£100 000 = £150 000
- Deprivation, measured using the Townsend Deprivation Index (TDI) of current address*
- Current employment status, coded as three separate outcomes
  - Non-employed, not retired (versus employed or retired)
  - Non-employed (versus employed, retired excluded)
  - Retired (versus still employed, other non-employed excluded)
- Job class, coded as skilled versus unskilled^22^
- Degree status, coded as degree-level education versus lower
- Owner-occupied accommodation versus renting

*Social Outcomes*

Measures of social contact

- Having someone to confide in weekly or more frequently versus less frequently
- Friend/family visits weekly or more frequently versus less frequently
- Cohabiting with partner or spouse versus not cohabiting
- Participation in any leisure/social activity versus none

Measures of happiness and well-being

- Lonely/isolated versus not lonely/isolated
- Extremely/very/moderately happy versus not
- Extremely/very/moderately happy with family relationship versus not
- Extremely/very/moderately happy with financial situation versus not
- Extremely/very/moderately happy with friendships versus not
- Extremely/very/moderately happy with health versus not
- Extremely/very/moderately happy with work/job versus not

*Household income and deprivation were both dichotomized as additional analyses so the results could be included in plots comparing across all outcomes: ≥£52 000 versus <£52 000 for household income, and most deprived third of TDI versus two least deprived thirds for deprivation.

### Main Mendelian randomization analysis

We used Mendelian randomization to estimate the causal association between each health condition and risk factor and each outcome, using the PRS as an instrumental variable, with age at baseline assessment, sex, UK Biobank recruitment centre and 40 genetic principal components as covariates. We used the ivreg2 package in Stata (version 15.1) with robust standard errors, and tested for weak instrument bias (using Kleibergen-Paap Wald rk F statistics) to assess whether the PRSs were sufficiently predictive of the exposures.^23^ This Mendelian randomization analysis estimates mean and risk differences for continuous and binary outcomes, respectively, using additive structural mean models.^24–26^ Mean differences are interpreted as the average change in the outcome over all participants for having the exposure, and risk differences are interpreted as the absolute percentage point change in proportion of participants with the outcome for having the exposure (as in a linear probability model). For health conditions, we are measuring the effects of genetic liability to the health condition.^27^ The analysis of breast cancer as an exposure was restricted to women. Despite the limitations of an approach based on statistical significance,^28^ the number of results generated in these analyses necessitated a decision about which results to present in the main paper. Therefore, in the main table of results, we report results with a *P*-value less than 0.0026 (a Bonferroni-corrected *P*-value of 0.05 divided by 19 outcomes, with no correction for multiple exposures), and full results are reported in Supplementary Tables, available as Supplementary data at *IJE* online. However, we considered the public health implications of all effect estimates when interpreting results.

To compare the Mendelian randomization results with associations from non-genetic analysis, we estimated the multivariable-adjusted associations between the exposures and outcomes using linear regression, with age, sex, recruitment centre and 40 genetic principal components as covariates, i.e. observational analyses without genetic variables. These are linear probability models for binary outcomes (rather than logistic regression models), which were necessary to be able to compare with the Mendelian randomization analyses, as they are equivalent to additive structural mean models. We also performed endogeneity tests^29^ to test whether the Mendelian randomization and multivariable-adjusted association estimates differed, where a low *P*-value indicates there was evidence that the Mendelian randomization and multivariable effects were different.

### Sensitivity analyses

The robustness of Mendelian randomization analyses is reliant on the assumption that the SNPs, and therefore PRSs, do not affect the outcome except through the exposure, i.e. the SNPs are not pleiotropic. We tested this assumption by conducting sensitivity Mendelian randomization analyses, including inverse-variance weighted (IVW), MR Egger (an indicator of directional pleiotropy), weighted median, weighted mode and simple mode analyses.^30–32^ We also measured Cochran’s Q statistic from the IVW analyses (a measure of heterogeneity in the effects of individual SNPs on the outcome), an indicator of pleiotropy^33^ or problems with modelling assumptions.^34^

From these analyses, we determined: (i) whether the results were consistent with the main Mendelian randomization analysis, which would indicate that the results of the main analysis were robust; and (ii) whether there was evidence of pleiotropy from both the Egger regression constant term and Cochran’s Q statistic. We also visually inspected plots of the sensitivity Mendelian randomization analyses, which would indicate possible bias in the results of the main analysis. Sensitivity Mendelian randomization analyses could only be performed when there were three or more SNPs included in each PRS.

We also conducted split-sample GWAS and Mendelian randomization analysis using UK Biobank data, in which we randomly split UK Biobank into halves, and for each half conducted a GWAS for each health condition and risk factor using the MRC IEU UK Biobank GWAS pipeline.^35^ The results of the two GWASs were used to create PRSs for the other half of UK Biobank avoiding sample overlap,^36^ and we repeated the Mendelian randomization analysis with the two PRSs separately, then combined the two results with fixed-effect meta-analysis to give a single estimate. The split-sample analysis: (i) allowed us to analyse lifetime smoking, as this has only been generated in UK Biobank, and thus no previous GWAS could have been used to inform the PRS; (ii) allowed us to potentially increase the size and power of the GWASs, possibly improving the predictive ability of the PRSs; and (iii) guaranteed homogeneity of the GWASs and analysis populations, which removes the potential bias from using data from an external GWAS to inform the creation of the PRSs, for example, through differences in populations giving different effects of SNPs. We also performed sensitivity Mendelian randomization sensitivity analyses on each split to check the robustness of the split-sample results.

Supplementary Table 5, available as Supplementary data at *IJE* online, shows a summary of all PRSs created and used in the split-sample analyses, and all GWAS significant SNPs from the split-sample GWASs are detailed in Supplementary Table 6, available as Supplementary data at *IJE* online.

### Secondary analyses

We conducted secondary analyses to check the robustness of results, looking at whether: (i) results are different by sex and deprivation at birth; (ii) results for household income are affected by household size (income equivalization); (iii) results for employment outcomes are different when restricting to working age participants; (iv) results for household income are different when restricting to participants who have not retired; and (v) results for smoking are robust when only looking at the SNP rs1051730, known to affect smoking heaviness.^37^ Additionally, we estimated the correlation between each of the PRSs in both the main analyses and within each split in the split-sample analyses, to determine whether any of the PRSs share genetic information. Further information for the secondary analyses and results are in Supplementary Information Section 4, available as Supplementary data at *IJE* online.

### Patient and public involvement

This study was conducted using UK Biobank. Details of patient and public involvement in the UK Biobank are available online [www.ukbiobank.ac.uk/about-biobank-uk/] and [https://www.ukbiobank.ac.uk/wp-content/uploads/2011/07/Summary-EGF-consultation.pdf? phpMyAdmin=trmKQlYdjjnQIgJ%2CfAzikMhEnx6]. No patients were specifically involved in setting the research question or the outcome measures, nor were they involved in developing plans for recruitment, design or implementation of this study. No patients were asked to advise on interpretation or writing up of results. There are no specific plans to disseminate the results of the research to study participants, but the UK Biobank disseminates key findings from projects on its website.

### Data and code availability

The empirical dataset will be archived with UK Biobank and made available to individuals who obtain the necessary permissions from the study’s data access committees. The code used to clean and analyse the data is available as Supplementary Materials at *IJE* online, and here: [https://github.com/sean-harrison-bristol/Effects-of-Health-Conditions-and-Risk-Factors-on-Socioeconomic-Outcomes].

## Results

Summary demographics, including prevalence of health conditions, risk factors and all outcomes, are presented in Table 1. The mean age of participants was 56.9 years (standard deviation: 8.0 years), mean household income (estimated from household income category midpoints) was £44 409 (standard deviation: £33 181) and 46% of participants were male. Results from the main Mendelian randomization analysis are displayed in a heat map of the *P*-values, where the *P*-value of each analysis is displayed in a cell, with the colour of the cell increasing in intensity as the *P*-value of the analysis decreases, Figure 1. Table 2 shows results from the main Mendelian randomization, split-sample Mendelian randomization and multivariable adjusted analyses for all outcomes where the main or split-sample Mendelian randomization analysis had a *P*-value less than 0.0026. All health conditions (except osteoarthritis) and risk factors in the main Mendelian randomization analysis had a low risk of weak instrument bias, and 75% of regressions had F statistics above 1000.

**Figure 2:**
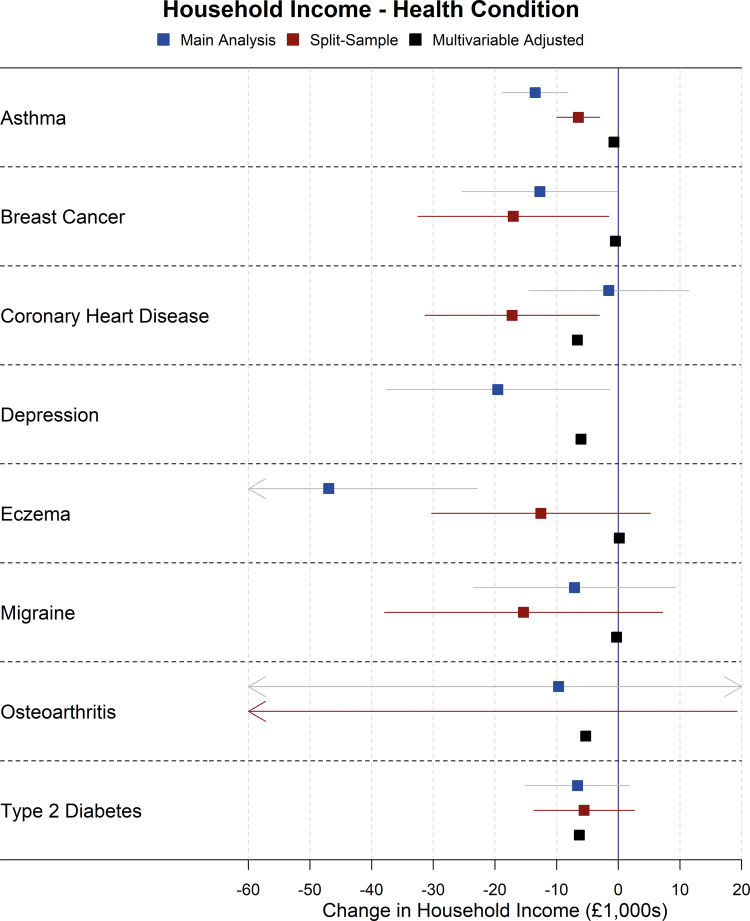
Forest plot showing effects of health conditions on household income for the main Mendelian randomization, split-sample Mendelian randomization and multivariable-adjusted analyses (note: confidence intervals are so narrow for the multivariable adjusted analyses that they cannot be seen).

**Figure 3:**
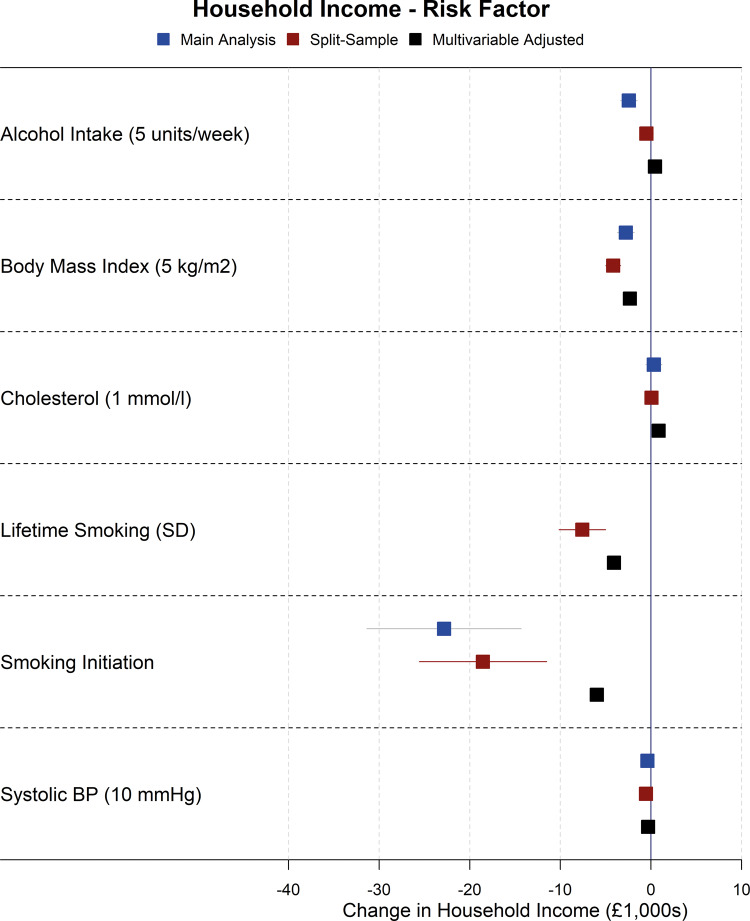
Forest plot showing effects of risk factors on household income for the main Mendelian randomization, split-sample Mendelian randomization and multivariable-adjusted analyses (note: confidence intervals are so narrow that they cannot be seen for most associations).

**Figure 4:**
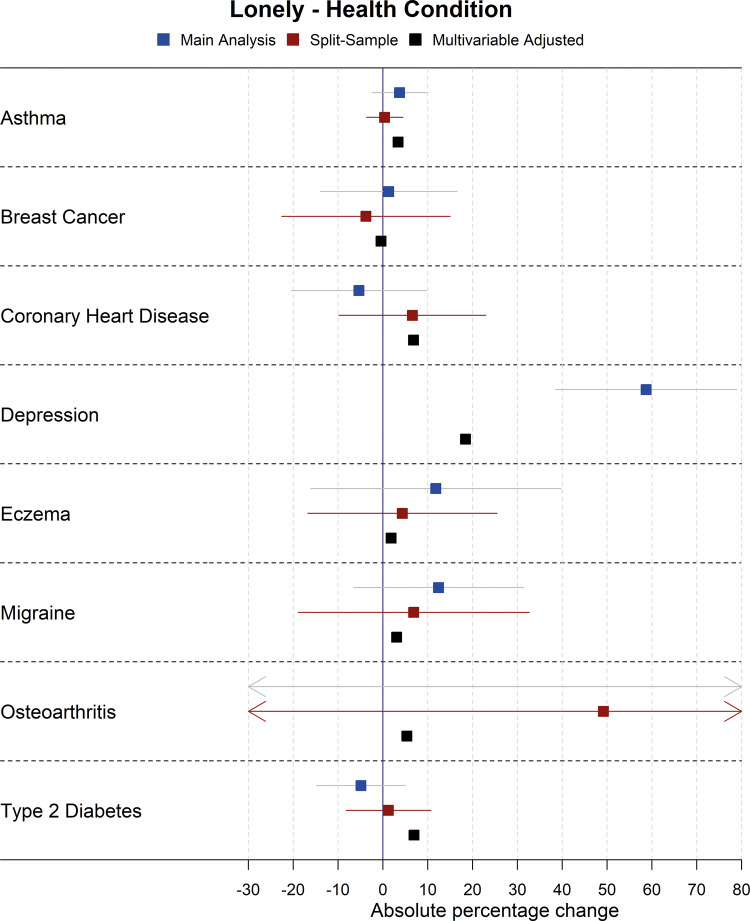
Forest plot showing effects of health conditions on being lonely for the main Mendelian randomization, split-sample Mendelian randomization and multivariable-adjusted analyses (note: confidence intervals are so narrow for the multivariable-adjusted analyses that they cannot be seen).

**Figure 5:**
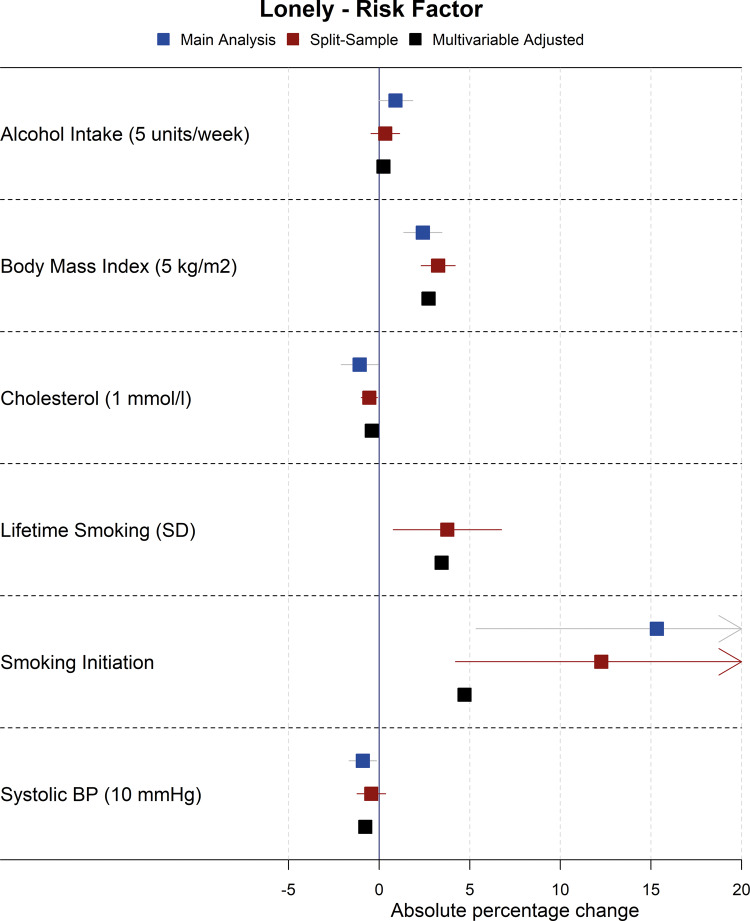
Forest plot showing effects of risk factors on being lonely for the main Mendelian randomization, split-sample Mendelian randomization and multivariable-adjusted analyses (note: confidence intervals are so narrow for the multivariable-adjusted analyses that they cannot be seen).

Forest plots showing the results for the main Mendelian randomization, split-sample Mendelian randomization and multivariable-adjusted analyses for health conditions and risk factors on household income are shown in Figures 2 and 3, although there was evidence of heterogeneity between SNPs in sensitivity Mendelian randomization analyses for some exposures on income (Cochran’s Q statistic *P* <0.01 for alcohol intake, BMI, breast cancer, depression, smoking initiation, systolic blood pressure), indicating possible pleiotropy. As such, results for income for these exposures should be interpreted with some caution, although there was little evidence of directional pleiotropy from MR Egger analyses of these exposures on income. As additional examples, the main Mendelian randomization, split-sample Mendelian randomization and multivariable-adjusted analyses for health conditions and risk factors on loneliness are shown in Figures 4 and 5; plots for all other analyses are presented in the Supplementary Materials, available as Supplementary data at *IJE* online.

### Health conditions

#### Asthma

In the main Mendelian randomization analysis, asthma was estimated to reduce household income [mean difference = -£13 474, 95% confidence interval (CI): -£18 749 to -£8199], the chance of obtaining a university degree [absolute percentage change [APC] = -17.1%, 95% CI: -25.4% to -8.7%] and the chance of cohabiting (APC = -11.0%, 95% CI: -17.9% to -4.0%). There was little evidence that asthma affected other outcomes. Split-sample Mendelian randomization analysis estimates similarly showed detrimental estimates of effects of asthma on obtaining a university degree and income, but not on cohabiting, and there was only evidence of pleiotropy in sensitivity Mendelian randomization analyses for obtaining a university degree. The multivariable-adjusted association estimates tended to be weaker than the Mendelian randomization estimates, and in some cases (e.g. the chance of obtaining a university degree) in the opposite direction.

#### Depression

In the main Mendelian randomization analysis, depression was estimated to reduce satisfaction with health (APC = -29.1%, 95% CI: -44.6% to -13.6%), financial situation (APC = -26.4%, 95% CI: -41.9% to -10.9%) and family relationships (APC = -19.3%, 95% CI: -30.4% to -8.1%) and, as expected, reduce the chance of being happy (APC = -19.1%, 95% CI: -28.4% to -9.8%) and increase the chance of being lonely (APC = 58.7%, 95% CI: 38.5% to 78.9%). CIs were wide, but the point estimates were consistent with depression being detrimental for almost all socioeconomic outcomes, including household income (mean difference = -£19 540, 95% CI: -£37 635 to -£1445). Depression was excluded from the split-sample analyses as no GWAS-significant SNPs were found in either split. There was evidence of heterogeneity in SNP effects for most outcomes, but no evidence of directional pleiotropy from Egger regression. Multivariable-adjusted association estimates tended to be weaker than Mendelian randomization estimates.

#### Eczema

In the main Mendelian randomization analysis, eczema was estimated to reduce household income (mean difference = -£46 965, 95% CI: -£71 028 to -£22 902). However, this was not observed in the split-sample Mendelian randomization analysis (mean difference = £-12 545, 95% CI: £-30 268 to £5177) or multivariable adjusted analysis (mean difference = £158, 95% CI: £-544 to £859). CIs for all other outcomes were very wide.

#### Migraine

In the main Mendelian randomization analysis, migraines were estimated to reduce the chance of having a weekly leisure or social activity (APC = -47.9%, 95% CI: -71.1% to -24.7%). This estimate was smaller in the split-sample Mendelian randomization (APC = -26.3%, 95% CI: -57.7% to 5.2%) and multivariable regression analyses (APC = -2.9%, 95% CI: -3.8% to -2.0%). When ‘Pub or social club’ was removed from the weekly leisure and social activity outcome, the main Mendelian randomization effect estimate was substantially reduced (APC = -23.4%, 95% CI: -47.9% to 1.2%), whereas looking only at going to a pub or social club weekly showed a stronger effect (APC = -68.5%, 95% CI: -90.8% to -46.1%). The CIs in Mendelian randomization analyses were wide for all other outcomes. There was no evidence of pleiotropy.

#### Type 2 diabetes

In the main and split-sample Mendelian randomization analyses, there were no strong associations for type 2 diabetes with any outcome. Directions of effects were inconsistent across outcomes. Multivariable-adjusted association estimates tended to be larger than Mendelian randomization estimates, and associations were apparent with several outcomes, most notably satisfaction with health (APC for multivariable adjusted association estimate = -19.1%, 95% CI: -20.1% to -18.2%).

#### Other health conditions

The CIs in Mendelian randomization analyses for breast cancer, coronary heart disease and osteoarthritis were very wide for all outcomes, and as such, these analyses were inconclusive. For breast cancer and coronary heart disease, there was no clear pattern of the direction of effects across outcomes, and CIs were wide. The CIs for osteoarthritis were very wide for all outcomes. As expected, given life course temporal relationships, there was little evidence from the main or split-sample Mendelian randomization analyses that breast cancer, coronary heart disease or osteoarthritis were associated with the chance of obtaining a university degree (included as negative controls). In the multivariable-adjusted analysis, breast cancer was not associated with the chance of obtaining a university degree, whereas coronary heart disease and osteoarthritis were (APC = -8.1%, 95% CI: -9.0% to -7.1% and APC = -6.3%, 95% CI: -6.9% to -5.6%, respectively), indicating, together with the null estimates from the Mendelian randomization analyses, possible social causation of the health conditions rather than vice versa. Osteoarthritis was excluded from the sensitivity Mendelian randomization analysis as there were fewer than three GWAS-significant SNPs in the osteoarthritis GWAS.

In the multivariable-adjusted analysis, breast cancer was only associated with increased chances of being non-employed and retired and a decreased satisfaction with health, whereas coronary heart disease and osteoarthritis were negatively associated with all economic outcomes and most social outcomes, though not satisfaction with friendships or work nor with weekly friend visits.

### Risk factors

#### Alcohol intake

All results are expressed for a 5 units per week increase in alcohol intake.

In the main Mendelian randomization analysis, alcohol was estimated to reduce household income (mean difference = -£2446, 95% CI: -£3362 to -£1530) and the chance of owning accommodation (APC = -1.8, -2.4% to -1.2%) and to increase deprivation (mean difference in TDI = 0.18, 95% CI: 0.11 to 0.25, approximately 23% of a decile of TDI). In the split-sample Mendelian randomization analysis, alcohol was estimated to reduce the chance of cohabiting (APC = -1.5%, 95% CI: -2.4% to -0.6%) and owning accommodation (APC = -1.2%, 95% CI: -1.7% to -0.6%) and to increase deprivation (mean difference in TDI = 0.14, 95% CI: 0.08 to 0.19, approximately 18% of a decile of TDI). There was no evidence of causal effects on other outcomes. There was evidence of heterogeneity in SNP effects for being happy, household income and receiving a university degree, but no evidence of directional pleiotropy in Egger regression. The multivariable-adjusted analysis estimated that alcohol increased (rather than reduced) household income (mean difference = £442, 95% CI: £400 to £484, *P*-value from endogeneity test = 1.6 x 10^-10^), and no associations were seen with other outcomes.

#### Body mass index

All results are expressed for a 5 kg/m^2^ increase in BMI.

In the main Mendelian randomization analysis, BMI was estimated to be detrimental for all socioeconomic outcomes. BMI was estimated to reduce household income (mean difference = -£2777, 95% CI: -£3692 to -£1863), and the chance of owning accommodation (APC = -1.6%, 95% CI: -2.4% to -0.8%), being satisfied with health (APC = -5.2%, 95% CI -6.8% to -3.5%), obtaining a university degree (APC = -2.9%, 95% CI: -4.4% to -1.5%) and having a skilled job (APC = -2.3%, 95% CI: -3.5% to -1.0%) and to increase deprivation (mean difference in TDI = 0.25, 95% CI: 0.17 to 0.33, approximately 31% of a decile of TDI) and the chance of being lonely (APC = 2.4%, 95% CI: 1.4% to 3.5%). In the split-sample analysis, effects of BMI were estimated to be more detrimental than in the main analysis for the above associations, and additionally to increase the chance of being non-employed, when both including and excluding retired participants (APC = 1.5%, 95% CI: 0.8% to 2.1% and APC = 2.3%, 95% CI: 1.3% to 3.2%, respectively), and to reduce the chance of being satisfied with financial situation (APC = -3.1%, 95% CI: -4.5% to -1.6%) and having a weekly leisure or social activity (APC = -3.0%, 95% CI: -4.2% to -1.9%).

There was evidence of heterogeneity in SNPs for most outcomes, but evidence of directional pleiotropy in Egger regression only for obtaining a university degree. The multivariable-adjusted associations between BMI and socioeconomic outcomes were generally consistent with the Mendelian randomization estimates.

### Cholesterol

All results are expressed for a 1-mmol/litre increase in cholesterol.

In the main and split-sample Mendelian randomization analyses, there was no evidence of effects of cholesterol on any outcome. In the multivariable-adjusted analyses, cholesterol was beneficial for all socioeconomic outcomes and most social contact and well-being outcomes. Together with the null estimates from the Mendelian randomization analyses, this could imply confounding or reverse causation in the multivariable-adjusted association estimates. Cholesterol was excluded from the sensitivity Mendelian randomization analysis as there were fewer than three GWAS-significant SNPs in the cholesterol GWAS.

#### Lifetime smoking

All results are expressed for a one standard deviation increase in the continuous lifetime smoking index value. We did not perform a main Mendelian randomization analysis, as there was no previous GWAS for lifetime smoking.

In the split-sample Mendelian randomization analysis, smoking was estimated to reduce household income (mean difference = -£7585, 95% CI: -£10 155 to -£5014), the chance of cohabiting (APC = -5.4%, 95% CI: -8.8% to -2.0%), owning accommodation (APC = -8.6%, 95% CI: -10.79% to -6.4%), having a skilled job (APC = -8.6%, 95% CI: -12.71% to -4.5%), obtaining a university degree (APC = -15.9%, 95% CI: -20.7% to -11.1%) and being satisfied with one’s financial situation (APC = -10.0%, 95% CI: -14.7% to -5.3%) and health (APC = -8.4%, 95% CI: -13.3% to -3.6%). Lifetime smoking was also estimated to increase deprivation (mean difference in TDI = 0.98, 95% CI: 0.76 to 1.19, approximately 123% of a decile of TDI) and the chance of being non-employed, with retired participants both included and excluded (APC = 4.2%, 95% CI: 2.1% to 6.2% and APC = 5.9%, 95% CI: 2.9% to 8.9% respectively). There was little evidence that smoking affected other social outcomes. There was evidence of heterogeneity in SNPs for obtaining a university degree, but no other outcomes, and no evidence of directional pleiotropy in Egger regression. Multivariable-adjusted analyses showed smaller estimates for all outcomes.

#### Smoking initiation

In the main Mendelian randomization analysis, smoking initiation was estimated to reduce household income (mean difference = -£22 838, 95% CI: -£31 354 to -£14 321), the chance of owning accommodation (APC = -20.8%, 95% CI: -28.2% to -13.4%), being satisfied with health (APC = -35.4%, 95% CI: -51.2% to -19.5%) and obtaining a university degree (APC = -65.9%, 95% CI: -81.4% to -50.4%), and to increase deprivation (mean difference in TDI = 1.73, 95% CI: 1.02 to 2.44, approximately 216% of a decile of TDI). All effects were also seen in the split-sample analysis. Smoking initiation was also estimated to increase the chance of having a skilled job (APC = -37.0%, 95% CI: -50.0% to -23.9%) and to reduce the chance of being non-employed, both including and excluding retired participants (APC = 13.3%, 95% CI: 6.3% to 20.2% and APC = 19.0%, 95% CI: 9.0% to 29.0%, respectively), and of having weekly friend visits (APC = 19.8%, 95% CI: 9.2% to 30.5%), but only in the main Mendelian randomization analysis. Additionally, smoking initiation was estimated to reduce the chance of being satisfied with one’s financial situation (APC = -22.7%, 95% CI: -36.0% to -8.9%) in the split-sample Mendelian randomization analysis, with a similar effect size in the main Mendelian randomization analysis. CIs were wide for all outcomes. There was evidence of heterogeneity in SNP effects for most outcomes, but no evidence of directional pleiotropy from Egger regression. Multivariable-adjusted association estimates tended to be closer to the null than the MR analyses.

#### Systolic blood pressure

All results are expressed for a 10-mmHg increase in systolic blood pressure (BP).

In the main and split-sample Mendelian randomization analyses, there was no evidence of effects of systolic BP on any outcome.

### Further analyses

Full results from main Mendelian randomization, sensitivity Mendelian randomization, split-sample Mendelian randomization and split-sample sensitivity Mendelian randomization analyses are shown in Supplementary Tables 7–10, available as Supplementary data at *IJE* online, respectively, with secondary and sensitivity analyses results in Supplementary Tables 11 and 12, available as Supplementary data at *IJE* online. For all health conditions and risk factors, forest plots showing results for the main Mendelian randomization, split-sample Mendelian randomization and multivariable-adjusted analyses (presented both as each exposure on social and socioeconomic outcomes, and for each outcome on health conditions and risk factors) are available in the Supplementary Materials, available as Supplementary data at *IJE* online, along with forest plots of SNPs and plots showing IVW, MR Egger, simple mode, weighted median and weighted mode Mendelian randomization analyses. There was little evidence of correlation between any PRSs in the main analysis (all R^2^ values below 0.01); however, for the split-sample PRSs, there was evidence of correlations between asthma and eczema (r = 0.17 for both splits combined), and between smoking initiation and lifetime smoking (r = 0.37 for both splits combined) (see Supplementary Table 13, available as Supplementary data at *IJE* online).

## Discussion

We estimate the putative causal effects of a variety of health conditions and risk factors on socioeconomic and social outcomes using Mendelian randomization, a genetically informed methodology typically less affected by confounding and reverse causality than observational analyses that adjust for measured confounders.^11^ Our results indicate that higher BMI, greater alcohol intake and smoking all negatively affect socioeconomic outcomes, and depression negatively affects many social outcomes. We do not observe an effect of cholesterol or systolic BP on any outcome, which may reflect effective treatments for high cholesterol and hypertension protecting participants from adverse consequences. For breast cancer, coronary heart disease, migraine and osteoarthritis, the confidence intervals for all Mendelian randomization analyses were all very wide, meaning that it is not possible to draw firm conclusions about the social and socioeconomic consequences of these conditions from our analyses. However, we estimated that migraine reduced the chance of going to a pub or social club weekly, possibly as alcohol increases the risk of migraines.^38^^,^^39^

Potential reasons for adverse effects of high BMI, alcohol use and smoking on social and socioeconomic outcomes include increased disease burden, social stigma (e.g. bias against obese people, smokers etc.) or behaviours which make employment, retention of employment or social interaction challenging. Our previous analyses of UK Biobank have shown evidence of effects of BMI on social and socioeconomic outcomes in both Mendelian randomization and non-genetic within-sibling analyses.^4^ Here, we build on these previous analyses by including a broader set of social and socioeconomic outcomes, conducting additional sensitivity and secondary analyses and facilitating comparisons across a range of health conditions and risk factors.

Higher genetic propensities towards asthma and eczema were estimated to reduce household income (mean difference = -£13 519, 95% CI: -£18 794 to -£8243 for asthma, and mean difference = -£46 987, 95% CI: -£71 048 to -£22 925 for eczema). However, it is possible that these estimates are susceptible to bias from pleiotropy, given the extreme size of the effects. Asthma and eczema share many genetic loci, along with inflammatory bowel disease and other autoimmune conditions.^40^ Therefore, the Mendelian randomization results for eczema and asthma may reflect an underlying genetic predisposition toward autoimmune condition susceptibility, rather than asthma or eczema specifically. This would not be detectable with Mendelian randomization sensitivity analyses if all SNPs included in the PRSs were affecting autoimmune susceptibility rather than the conditions themselves (directional unbalanced pleiotropy). Additionally, the PRS for smoking initiation may capture impulsivity and risk taking as well as a propensity to smoke.

For some health conditions (asthma, breast cancer, eczema, migraine), we saw little evidence for observational (multivariable-adjusted) associations with either socioeconomic or social outcomes, despite previous evidence often showing strong associations. For example, breast cancer has been associated with lower income,^41^ but there was no observational association between breast cancer and household income in UK Biobank. This could result from selection bias in UK Biobank,^42^ with participants potentially liable to have less severe/advanced forms of the condition or quicker recovery than all breast cancer patients across a population, and also to have greater financial support and better employment conditions than the general population. The effects of health conditions may also diminish over time; there is some evidence that the negative effect on income among breast cancer survivors reduces over time.^41^ It is therefore possible that our study does not have the correct time frame to capture the effects of each health condition, or that well-functioning insurance markets and pension provision could mitigate socioeconomic effects of health conditions, at least within this generally affluent UK population.^43^ Additionally, if a participant developed any health condition after baseline, we would only know if the participant had a hospital episode which mentioned the condition.

There was evidence that depression was detrimental to multiple social outcomes, including reduced happiness and reported satisfaction rates and increased loneliness. Given these are common features of depression, this result was expected and gives us confidence that the PRS for depression was suitably predictive of depression.

### Strengths and limitations

The main strengths of this analysis are that Mendelian randomization analyses are generally less affected by confounding and reverse causation than multivariable-adjusted (observational) analyses,^44^ and that UK Biobank is a very large sample with sufficient data to enable us to examine multiple health exposures and multiple socioeconomic and social outcomes. For some associations, there were marked differences between the Mendelian randomization and multivariable-adjusted association estimates, which could result from reverse causation or confounding in the multivariableassociation adjusted estimates. For example, coronary heart disease was associated with a decreased chance of obtaining a university degree (APC = -8.1%, 95% CI: -9.0% to -7.1%) in the multivariable-adjusted analysis, which is implausible given that coronary heart disease usually occurs later in life than attending university, and this association was not seen in the Mendelian randomization analysis. Additionally, the SNPs contributing to the PRSs were drawn from GWASs that excluded UK Biobank to avoid biases caused by sample overlap,^36^ and all reached genome-wide significance. Finally, the results from the main and split-sample analyses were largely consistent across exposures and outcomes, reducing the possibility of bias from differences in SNP effects between the GWAS and UK Biobank populations.

However, Mendelian randomization rests on assumptions that cannot be proven to be true.^44^ Assessing pleiotropy was difficult or impossible for many exposures, due to the low number of SNPs and wide CIs, but there was evidence for heterogeneity between SNPs for some associations (e.g. for income), and directional pleiotropy from Egger regression for a limited number of associations (e.g. for BMI on obtaining a university degree). As the outcomes were social and socioeconomic, not biological, the exclusion restriction assumption would be strong for any genetic variant (i.e. that the genetic variant affects the outcome only through the exposure). For example, we cannot assume that an SNP associated with income affects any health condition or risk factor solely through income. We therefore did not perform bidirectional Mendelian randomization,^45^ and so cannot rule out reverse causation for any analysis.

The PRSs represent lifetime exposure to or risk for the health condition or risk factor, and interventions to reduce the exposure or risk of the exposure at different time points in a person’s life may have different effects; effects at specific points in life cannot be explored with the methodology used in this paper. As we used linear prediction models for all analyses, some effect estimates may also be impossibly large (i.e. over 100%), which could occur when precision is very low, though this was rare. Although Mendelian randomization is generally less affected by confounding and reverse causality than multivariable regression analyses, an important potential source of bias in these analyses is family-level effects. Recent evidence suggests that assortative mating and dynastic effects can lead to bias in Mendelian randomization effect estimates,^46^ with estimates of the effect of BMI on educational attainment being consistent with the null in within-family Mendelian randomization models using data from UK Biobank and the Norwegian HUNT study. In our previous analysis of UK Biobank,^4^ within-family Mendelian randomization models in UK Biobank alone were too imprecise to draw conclusions about whether the estimated effects of BMI on social and socioeconomic outcomes are robust to potential confounding by family-level factors. Since BMI is the exposure for which we have greatest statistical power (due to the strength of the genetic instrumental variable), we have not repeated the within-family analyses for our other exposures, as power will be extremely limited. However, as more datasets are available that include genetic information for multiple family members, examination of whether these effects can be detected with a within-family Mendelian randomization design will be a high priority.

UK Biobank, although large, is not representative of the UK population as participants tend to be wealthier and healthier compared with the country as a whole, which may impart bias to our analyses.^47^ It is likely that this biased some estimates towards the null, as wealthier and healthier people may be more resistant to any detrimental effects of health conditions and risk factors. Additionally, there is evidence of a geographical structure in the UK Biobank genotype data which cannot be accounted for using adjustment for principal components, which may also have biased our analyses.^48^ However, recent evidence suggests that whereas geographical structure may be present after controlling for principal components in the PRSs for BMI, coronary heart disease, smoking and alcohol consumption (and these may all be related to educational attainment), there was little evidence for geographical structure in the PRSs for other health conditions.^49^ Additionally, a recent GWAS of income showed that only 8% of the inflation in the GWAS test statistics was due to residual stratification or confounding, indicating that population structure is unlikely to severely bias many of our results.^50^ Some outcomes were dichotomized, which may have reduced our ability to detect associations (e.g. satisfaction with health).

For health conditions, the uncertainty around the Mendelian randomization effect estimates was large. As many health conditions had small associations with outcomes on multivariable-adjusted analyses, this often meant the Mendelian randomization estimates were larger than the observed estimates or had a different sign, but this can be explained by the imprecision in the Mendelian randomization estimates. The uncertainty is due in part to the relatively poor ability of the PRS to predict some health conditions. There were minimal differences in prevalence between UK Biobank and the UK for most health conditions studied (apart from migraine and depression, which were less and more prevalent in UK Biobank respectively), but it is possible the health conditions were milder or better managed in UK Biobank participants compared with the population as a whole.^51^ Therefore, null results should be interpreted as a lack of evidence for a causal effect, not evidence of a lack of a causal effect.

## Conclusion

The results of this study imply that higher BMI, smoking and alcohol consumption are likely detrimental to socioeconomic outcomes. Whereas the prevalence of smoking is decreasing in the UK,^52^ the average BMI has risen and is continuing to rise worldwide.^53^ Reducing average BMI levels, and further reducing smoking and alcohol intake, in addition to health benefits may also improve socioeconomic outcomes for individuals and populations.

There was little evidence of causal effects of health conditions on socioeconomic outcomes, which may reflect true absence of causal effects or bias due to the characteristics of UK Biobank participants, or the low precision of our estimates for health condition effects.

## Supplementary data

Supplementary data are available at *IJE* online.

## Funding

This work was supported by the Health Foundation as part of a project entitled ‘Social and Economic Consequences of Health: Causal Inference Methods and Longitudinal, Intergenerational Data’, which is part of the Health Foundation’s Social and Economic Value of Health programme (Grant ID: 807293). L.D.H. is funded by a Career Development Award from the UK Medical Research Council (MR/M020894/1). M.G., S.V.K. and D.C. work for the Health Foundation project entitled ‘Causal Effects of Alcohol and Mental Health Problems on Employment Outcomes: Harnessing UK Biobank and Linked Administrative Data’. The Health Foundation is an independent charity committed to bringing about better health and health care for people in the UK. The Medical Research Council (MRC) and the University of Bristol support the MRC Integrative Epidemiology Unit [MC_UU_12013/1, MC_UU_12013/9, MC_UU_00011/1]. The Economics and Social Research Council (ESRC) support N.M.D. via a Future Research Leaders grant [ES/N000757/1] and a Norwegian Research Council Grant number 295989. P.D. acknowledges support from an MRC Skills Development Fellowship (MR/P014259/1). S.V.K. acknowledges funding from an NHS Research Scotland Senior Clinical Fellowship (SCAF/15/02). The MRC/CSO Social & Public Health Sciences Unit, University of Glasgow is supported by the Medical Research Council (MC_UU_12017/13 & MC_UU_12017/15) and the Scottish Government Chief Scientist Office (SPHSU13 & SPHSU15). H.E.J. acknowledges support from an MRC Career Development Award in Biostatistics (MR/M014533/1).

## Supplementary Material

dyaa114_supplementary_data
