# Supplementary material for: The causal effects of health conditions and risk factors on social and socioeconomic outcomes: Mendelian randomization in UK Biobank
Source: Int J Epidemiol. 2020 Aug 18;49(5):1661–81. doi: 10.1093/ije/dyaa114 (PMC7746412; doi:10.1093/ije/dyaa114)
Supplement: dyaa114_supplementary_data [file dyaa114_supplementary_data.zip › ije-2019-11-1552-File013.docx]

# Supplementary Information

## 1. Inclusion Criteria and Genotyping

### Inclusion Criteria

We restricted analyses to individuals of white British ancestry, as defined by participants who self-reported as “White British” and who had very similar ancestral backgrounds according to the principal component analysis (n=409,703), as described by Bycroft (1). We excluded individuals with sex-mismatch (derived by comparing genetic sex and reported sex) or individuals with sex-chromosome aneuploidy from the analysis (n=814). We estimated kinship coefficients using the KING toolset (2) and identified 107,162 pairs of related individuals (1). We applied an in-house algorithm to this list and preferentially removed the individuals related to the greatest number of other individuals until no related pairs remain. This resulted in the exclusion of 79,448 individuals. Additionally, two individuals were removed due to them relating to a very large number (>200) of individuals, and 135 individuals were excluded as they withdrew from the study. After exclusions, 336,997 participants remained.

### Genotyping

The full data release contains the cohort of successfully genotyped samples (n=488,377). 49,979 individuals were genotyped using the UK BiLEVE array and 438,398 using the UK Biobank axiom array. Pre-imputation QC, phasing and imputation are described elsewhere (1). In brief, prior to phasing, multiallelic SNPs or those with MAF ≤1% were removed. Phasing of genotype data was performed using a modified version of the SHAPEIT2 algorithm (3). Genotype imputation to a reference set combining the UK10K haplotype and HRC reference panels (4) was performed using IMPUTE2 algorithms (5). The analyses presented here were restricted to autosomal variants within the HRC site list using a graded filtering with varying imputation quality for different allele frequency ranges. Therefore, rarer genetic variants are required to have a higher imputation INFO score (Info>0.3 for minor allele frequency (MAF) >3%; Info>0.6 for MAF 1-3%; Info>0.8 for MAF 0.5-1%; Info>0.9 for MAF 0.1- 0.5%) with MAF and Info scores having been recalculated on an in house derived ‘European’ subset.

Further information on the MRC-IEU quality control of UK Biobank genetic data is available online (6).

## 2. Genome Wide Association Study Search and Polygenic Risk Score Generation

We searched MR-Base (7) and the NHGRI-EBI Catalog of published genome-wide association studies (GWAS) (<https://www.ebi.ac.uk/gwas>) to find suitable GWAS. If we found no suitable GWAS for any health condition or risk factor, we conducted an online search for a previous GWAS. Only European populations were considered, and if we found multiple GWAS for the same health condition or risk factor, we selected the GWAS with the most participants. We searched for proxy SNPs with an R^2^ above 0.8 (using genotype data from European individuals (CEU) from phase 3 (version 5) of the 1000 Genomes project (8)) for any SNP missing from UK Biobank. SNPs from each GWAS were clumped using an R^2^ threshold of 0.001 and a window of 10,000 kilo-bases.

We selected GWAS-significant SNPs for smoking initiation and alcohol intake from GSCAN (9), excluding the UK Biobank and 23andMe datasets.

To estimate the strength of the association between the PRS and the health condition or risk factor it proxied, we regressed each health condition or risk factor against its PRS using linear or logistic regression (as appropriate) with no covariables to estimate the R^2^ (or pseudo-R^2^) value.

The Wray et al. GWAS (2018 (11)) included the pilot sample of UK Biobank, and therefore we only created a PRS for participants not in the pilot sample (241,868 of 336,997 participants, 72%), to preserve independence of the GWAS and analysis samples. This reduced the number of participants with a depression phenotype and PRS to 94,131 of the potential 336,997 participants (28%).

There was no previous GWAS for lifetime smoking, as this was only measured in UK Biobank. As such, lifetime smoking was not considered in the main Mendelian randomization analysis, only in the split-sample Mendelian randomization analysis.

## 3. Outcome Definitions

### Household Income and Deprivation

Household income was recoded from categories to numerical values taking the mid-point of the range, or a nominal value for the open-ended categories, and analysed as a continuous variable:

- <£18,000 = £15,000
- £18,000 to £30,999 = £24,500
- £31,000 to £51,999 = £41,500
- £52,000 to £100,000 = £76,000
- >£100,000 = £150,000

As household income and deprivation (measured using the Townsend Deprivation Index [TDI]) were the only continuous outcomes, we created binary variables for both to allow for comparison between all outcomes, especially on plots. For household income, we compared those with a total household income above and below £52,000 (i.e. upper two categories of household income versus bottom three categories). For deprivation, we split the participants into tertiles of TDI, and compared the most deprived tertile with the remaining two tertiles. These results are included in forest plots of results, but not included in **Table 2**.

### Employment

Job class was coded as skilled versus unskilled as in the Tyrrell (2016, (12)) Mendelian randomization analysis of height, BMI and socioeconomic status, where a skilled job was defined as ones in the following categories:

1. Managers and Senior Officials
2. Professional Occupations
3. Associate Professional and Technical Occupations
4. Administrative and Secretarial Occupations
5. Skilled Trades Occupations

Unskilled jobs were defined as ones in the following categories:

1. Personal Service Occupations
2. Sales and Customer Service Occupations
3. Process, Plant and Machine Operatives
4. Elementary Occupations

Current employment status was coded as:

1. Non-employed, not retired (versus employed or retired)
2. Non-employed (versus employed, retired excluded)
3. Retired (versus still employed, other non-employed excluded)

### Degree Status

Degree status was coded as having a college or university degree. We did not consider professional qualifications to be equivalent to degree-level education.

### Social Outcomes

We dichotomised the frequency of confiding and friend/family visits, comparing weekly or more frequently with less frequently.

We dichotomised all satisfaction outcomes, comparing satisfied (extremely/very/moderately happy) with not satisfied (extremely/very/moderately unhappy).

We defined cohabiting with partner as positive if the participant’s response to the question, "How are the other people who live with you related to you?" included “husband, wife or partner”.

## 4.1 Secondary Analyses

### Interactions with Sex and Deprivation at Birth

As both sex and deprivation at birth could modify the effect of health conditions and risk factors on outcomes, we repeated the main Mendelian randomization analyses separately in men and women and within thirds of TDI at birth. We then compared the stratum-specific estimates to determine whether there was statistical evidence of differences by sex or between the top and bottom thirds of TDI at birth (13). We considered any P value lower than 0.01 to be indicative of an interaction.

Current deprivation of place of birth was estimated from east and north co-ordinates of birthplace and the Index of Multiple Deprivation taken from the February 2017 Office of National Statistics Postcode Directory (<http://ons.maps.arcgis.com/home/item.html?id=dfa0ff74981b4b228d2030d852f0b14a>) (17). Co-ordinates of birth were matched with the nearest postcode, although UK Biobank rounded the co-ordinates, so matching is not necessarily precise.

This approach has the strong assumption that the deprivation of a postcode at the birth of a participant is equivalent to the deprivation of the same postcode in 2017, and also that postcode of place of birth has been accurately recorded and is a reliable proxy for deprivation of the participant at birth. As such, the results from this analysis should be treated with caution.

### Results – Interactions with Sex

**Supplementary Table S11** contains all results for interactions from secondary analyses.

There was evidence of an interaction of migraine and sex on the outcome of having a weekly leisure or social activity (females: APC = -22.1%, 95% confidence interval [CI]: -43.4% to -0.7%; males: APC = -141%, 95% CI: -219% to -63.6%, P for difference = 0.0037). When “Pub or social club” was removed from the weekly leisure and social activity outcome, evidence for an interaction was substantially reduced (females: APC = -15.5%, 95% CI: -38.1% to 7.0%; males: APC = -49.6%, 95% CI: -129% to 30.2%, P for difference = 0.42). However, when going to a pub or social club weekly was the outcome, there was stronger evidence of an interaction of migraine and sex (females: APC = -31.3%, 95% CI: -49.7% to -12.9%; males: APC = -205%, 95% CI: -291% to -120%, P for difference = 0.000094).

There was also evidence of an interaction of BMI and sex on the outcome of cohabiting (females, difference for a 5 kg/m^2^ increase in BMI = -2.9%, 95% CI: -4.5% to -1.3%; males = 0.6%, 95% CI: -1.2% to 2.5%, P for difference = 0.0047). Additionally, there was evidence of an interaction of systolic blood pressure and sex on the outcome of being happy (females, difference for a 10 mmHg increase in systolic blood pressure = -0.2%, 95% CI: -1.0% to 0.6%; males = 2.0%, 95% CI: 0.6% to 3.5%, P for difference = 0.0063).

There was little evidence of interactions by sex for other associations.

### Results – Interactions with Deprivation at Birth

There was little evidence of interactions by deprivation at birth for any association.

### Correlations of PRS

We estimated the correlations of all PRS used in the main analysis, as well as within each split of the split-sample analyses, to determine whether there was evidence of shared genetic information being used in multiple PRS. This would indicate, for instance, whether there was evidence that the PRS for smoking and alcohol intake shared genetic information, for example through a propensity towards risk-taking behaviour. For the main analysis, we used simple correlation. For the split-sample analysis, we used simple correlation within each split, then combined the results using fixed-effect meta-analysis. The results for the main analysis, each split and the splits combined is presented in **Supplementary Table S13**. We considered a correlation coefficient of 0.1 (corresponding to an R^2^ of 0.01) or higher to indicate the presence of an association between the PRS.

### Results – Correlations of PRS

There was little evidence of associations between any PRS in the main analysis.

There was evidence in the split-sample analysis for correlations between asthma and eczema (correlation coefficient = 0.17 for both splits combined), and, as expected, between smoking initiation and lifetime smoking (correlation coefficient = 0.37 for both splits combined). However, we detected no other associations.

These results indicate we have little evidence that the PRS we used share genetic information, except for asthma and eczema.

## 4.2 Sensitivity Analyses

**Supplementary Table S12** contains all results for sensitivity analyses.

### Equivalisation of Household Income

We equivalised household income before tax (household income divided by the number in household) to explore whether household size contributed to effects on household income.

In the main Mendelian randomization analysis, alcohol intake, asthma, BMI, eczema and smoking initiation were estimated to reduce household income. The following health conditions and risk factors were all estimated to reduce equivalised household income: asthma (mean difference = -£4,265, 95% CI: -£6,831 to -£1,699), eczema (mean difference = -£17,533, 95% CI: -£29,070 to -£5,997), smoking initiation (mean difference = -£10,063, 95% CI: -£14,190 to -£5,936) and alcohol intake (mean difference for a 5 unit increase = -£755, 95% CI: -£1,193 to -£318). BMI (mean difference for a 5 kg/m^2^ increase = -£575, 95% CI: -£1,020 to -£130) was still estimated to be detrimental to equivalised household income, but with a larger P value. No other health condition or risk factor was estimated to materially affect equivalised household income.

These results indicate household size may account for some of the estimated effect of BMI on household income, though BMI was still estimated to be detrimental to equivalised household income.

### Restriction of Household Income to Participants Who Have Not Retired

We restricted household income to participants who had not retired to explore whether retirement contributed to effects on household income

In the main Mendelian randomization analysis, alcohol intake, asthma, BMI, eczema and smoking initiation were estimated to reduce household income. When restricted to participants who had not retired, alcohol intake (mean difference for a 5 unit increase = -£2,566, 95% CI: -£3,777 to -£1,356), asthma (mean difference = -£13,842, 95% CI: -£20,813 to -£6,870), BMI (mean difference for a 5 kg/m^2^ increase = -£2,806, 95% CI: -£4,054 to -£1,558), and smoking initiation (mean difference = -£25,464, 95% CI: -£37,888 to -£13,040) were all estimated to reduce equivalised household income with P values below 0.0026. Eczema (mean difference = -£36,346, 95% CI: -£64,000 to -£8,692) was still estimated to be reduce household income with a similarly large effect to the main Mendelian randomization analysis, but with a larger P value. No other health condition or risk factor was estimated to materially change equivalised household income.

These results indicate that early retirement did not account for the estimated effects of health conditions and risk factors on household income.

### Restriction of Employment Outcomes to Working Age

We restricted current employment status outcomes to participants of less than 65 years of age to explore whether early retirement contributed to effects on employment.

In the main Mendelian randomization analysis, smoking initiation was estimated to increase the chance of being non-employed versus employed, both with and without retired participants included in the analysis. When restricted to participants of working age, smoking initiation was still estimated to reduce the chance of being non-employed, both including and excluding retired participants (APC = 16.9%, 95% CI: 8.3% to 25.5% and APC = 20.1%, 95% CI: 9.7% to 30.6% respectively).

These results indicate that early retirement did not account for the estimated effect of smoking initiation on employment outcomes.

### Smoking Heaviness SNP: rs1051730

We analysed the effect of rs1051730 on all outcomes to examine whether the effects of lifetime smoking were replicated, as smoking SNPs may be pleiotropic, for instance for SNPs affecting impulsivity. Rs1051730 is a SNP in the nicotinic acetylcholine receptor alpha 3 subunit CHRNA3 gene, and has been used as a conservative proxy for smoking heaviness (14). We analysed subgroups to better estimate the effects of the SNP, analysing: 1) all participants, 2) ever smokers, 3) current smokers, 4) former smokers, and 5) never smokers. Analyses were conducted for all outcomes with rs1051730 as the exposure using linear and logistic regression (as appropriate), and with rs1051730 as an instrumental variable and lifetime smoking (for all participants and those that have smoked) or smoking initiation (for all participants only) as exposures in Mendelian randomization analysis.

### rs1051730 as a Proxy for Lifetime Smoking

In the split-sample Mendelian randomization analysis, lifetime smoking was estimated to reduce household income, the chance of cohabiting, owning accommodation, having a skilled job, receiving a university degree, and being satisfied with one’s financial situation and health, and increase deprivation and the chance of being lonely and being non-employed, both with retired participants included and excluded.

There were no estimates with P values below 0.0026 when using rs1051730 as an instrumental variable for lifetime smoking in any group of participants (all, ever smokers, current smokers, former smokers). However, CIs were very wide, and some effect sizes were of a similar magnitude to the main Mendelian randomization analysis. For example, lifetime smoking was estimated to reduce household income in all groups (e.g. mean difference for ever smokers = -£5,800, 95% CI: -£10,984 to -£618) and the chance of cohabiting in all groups (e.g. APC for ever smokers = -4.1%, 95% CI: -11.7% to 3.5%). However, other effects were estimated to be inconsistent with the main Mendelian randomization analysis, for example lifetime smoking was estimated to reduce deprivation in subgroups of smokers using rs1051730 as the instrumental variable (mean difference for ever smokers = -0.33, 95% CI: -0.86 to 0.19, approximately 41% of a decile of TDI).

### rs1051730 as a Proxy for Smoking Initiation

In the main and split-sample Mendelian randomization analyses, smoking initiation was estimated to reduce household, the chance of owning accommodation, being satisfied with health, and of receiving a university degree, and increase deprivation.

There were no estimates with P values below 0.0026 using rs1051730 as an instrumental variable for smoking initiation in all participants. However, CIs were very wide, and most effect sizes were of a similar magnitude to the main Mendelian randomization analysis. For example, smoking initiation was estimated to reduce household income (mean difference = -£15,415, 95% CI: -£33,693 to £2,863) and increase deprivation (mean difference in TDI = 0.85, 95% CI: -0.73 to 2.43, approximately 106% of a decile of TDI).

### rs1051730 Alone

There were no associations with P values below 0.0026 in any group of participants (all, ever smokers, current smokers, never smokers, former smokers) between rs1051730 and any outcome when analysed using linear or logistic regression (as appropriate). However, CIs were very wide. When restricting to P values of less than 0.05, rs1051730 was estimated to increase satisfaction with friendship in current smokers (APC = 23.1%, 95% CI: 5.6% to 40.6%) and ever smokers (APC = 9.2%, 95% CI: 0.1% to 18.4%), reduce household income in current smokers (mean difference = -£572, 95% CI: -£1,082 to -£62) and ever smokers (mean difference = -£286, 95% CI: -£566 to -£6), and reduce weekly friend visits in never smokers (APC = -1.7%, 95% CI: -3.2% to -0.2%).

Overall, the estimates from sensitivity analyses using rs1051730 have CIs that are too wide to confirm whether the main and split-sample Mendelian randomization estimates for lifetime smoking and smoking initiation are driven by smoking heaviness or other factors. However, many of the estimates using rs1051730 are consistent with the estimates using the PRS for lifetime smoking and smoking initiation, increasing our confidence in those results, especially for household income.

# References

1. Bycroft C, Freeman C, Petkova D, Band G, Elliott LT, Sharp K, et al. Genome-wide genetic data on ~500,000 UK Biobank participants. bioRxiv [Internet]. 2017;166298. Available from: https://www.biorxiv.org/content/early/2017/07/20/166298

2. Manichaikul A, Mychaleckyj JC, Rich SS, Daly K, Sale M, Chen WM. Robust relationship inference in genome-wide association studies. Bioinformatics. 2010;26(22):2867–73.

3. O’Connell J, Sharp K, Shrine N, Wain L, Hall I, Tobin M, et al. Haplotype estimation for biobank-scale data sets. Nat Genet. 2016;48(7):817–20.

4. Huang J, Howie B, McCarthy S, Memari Y, Walter K, Min JL, et al. Improved imputation of low-frequency and rare variants using the UK10K haplotype reference panel. Nat Commun. 2015;6.

5. Howie B, Marchini J, Stephens M. Genotype Imputation with Thousands of Genomes. G3&amp;#58; Genes|Genomes|Genetics [Internet]. 2011;1(6):457–70. Available from: http://g3journal.org/lookup/doi/10.1534/g3.111.001198

6. Mitchell, R., Hemani, G., Dudding, T., Corbin, L., Harrison, S., Paternoster L. UK Biobank Genetic Data: MRC-IEU Quality Control, version 2 - Datasets - data.bris [Internet]. data.bris. 2018. Available from: https://data.bris.ac.uk/data/dataset/1ovaau5sxunp2cv8rcy88688v

7. Hemani G, Zheng J, Elsworth B, Wade KH, Haberland V, Baird D, et al. The MR-Base platform supports systematic causal inference across the human phenome. Elife [Internet]. 2018;7:e34408. Available from: https://elifesciences.org/articles/34408

8. Altshuler DM, Durbin RM, Abecasis GR, Bentley DR, Chakravarti A, Clark AG, et al. An integrated map of genetic variation from 1,092 human genomes. Nature. 2012;135(V):0–9.

9. Liu M, Jiang Y, Wedow R, Li Y, Brazel DM, Chen F, et al. Association studies of up to 1.2 million individuals yield new insights into the genetic etiology of tobacco and alcohol use. Vol. 51, Nature Genetics. 2019. p. 237–44.

10. Okbay A, Baselmans BML, De Neve JE, Turley P, Nivard MG, Fontana MA, et al. Genetic variants associated with subjective well-being, depressive symptoms, and neuroticism identified through genome-wide analyses. Nat Genet. 2016;48(6):624–33.

11. Wray NR, Ripke S, Mattheisen M, Trzaskowski M, Byrne EM, Abdellaoui A, et al. Genome-wide association analyses identify 44 risk variants and refine the genetic architecture of major depression. Nat Genet. 2018;50(5):668–81.

12. Tyrrell J, Jones SE, Beaumont R, Astley CM, Lovell R, Yaghootkar H, et al. Height, body mass index, and socioeconomic status: Mendelian randomisation study in UK Biobank. BMJ. 2016;352.

13. Altman DG, Bland JM. Interaction revisited: The difference between two estimates. BMJ. 2003;326(7382):219.

14. Thorgeirsson TE, Geller F, Sulem P, Rafnar T, Wiste A, Magnusson KP, et al. A variant associated with nicotine dependence, lung cancer and peripheral arterial disease. Nature. 2008;452(7187):638–42.
