# Supplementary material for: The causal effects of health conditions and risk factors on social and socioeconomic outcomes: Mendelian randomization in UK Biobank
Source: Int J Epidemiol. 2020 Aug 18;49(5):1661–81. doi: 10.1093/ije/dyaa114 (PMC7746412; doi:10.1093/ije/dyaa114)
Supplement: dyaa114_supplementary_data [file dyaa114_supplementary_data.zip › ije-2019-11-1552-File014.docx]

Supplementary Figure


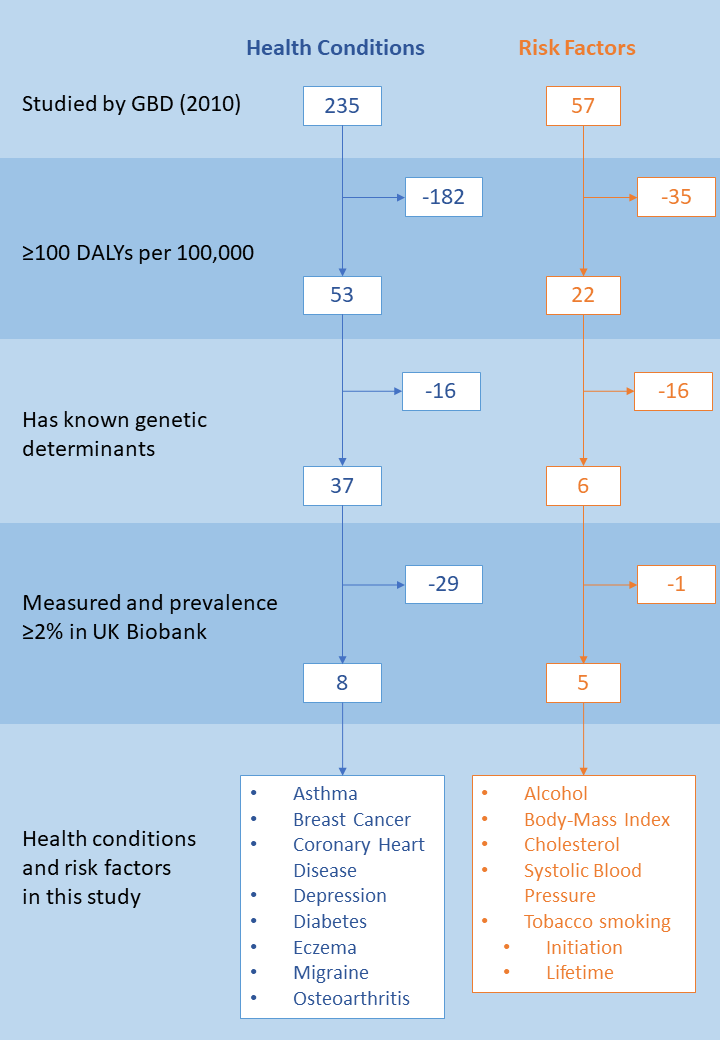


**Supplementary** **Figure S1**: Flow chart showing how health conditions and risk factors were chosen for inclusion in this study
